# The Role of Caspase-12 in Retinal Bystander Cell Death and Innate Immune Responses against MCMV Retinitis

**DOI:** 10.3390/ijms22158135

**Published:** 2021-07-29

**Authors:** Xinyan Zhang, Jinxian Xu, Brendan Marshall, Zheng Dong, Sylvia B. Smith, Ming Zhang

**Affiliations:** 1 Department of Cellular Biology and Anatomy, Medical College of Georgia, Augusta University, Augusta, GA 30912, USA; XZHANG1@augusta.edu (X.Z.); Jinxu@augusta.edu (J.X.); BMARSHALL@augusta.edu (B.M.); zdong@augusta.edu (Z.D.); sbsmith@augusta.edu (S.B.S.); 2 James and Jean Vision Discovery Institute, Medical College of Georgia, Augusta University, Augusta, GA 30912, USA; 3 Charlie Norwood VA Medical Center, Augusta, GA 30904, USA; 4 Department of Ophthamology, Medical College of Georgia, Augusta University, Augusta, GA 30912, USA

**Keywords:** caspase-12, murine cytomegalovirus, interferons, p53, caspase-3

## Abstract

(1) Background: caspase-12 is activated during cytomegalovirus retinitis, although its role is presently unclear. (2) Methods: *caspase-12^−/−^* (KO) or *caspase-12^+/+^* (WT) mice were immunosup eyes were analyzed by plaque assay, TUNEL assay, immunohistochemical staining, western blotting, and real-time PCR. (3) Results: increased retinitis and a more extensive virus spread were detected in the retina of infected eyes of KO mice compared to WT mice at day 14 p.i. Compared to MCMV injected WT eyes, mRNA levels of interferons α, β and γ were significantly reduced in the neural retina of MCMV-infected KO eyes at day 14 p.i. Although similar numbers of MCMV infected cells, similar virus titers and similar numbers of TUNEL-staining cells were detected in injected eyes of both KO and WT mice at days 7 and 10 p.i., significantly lower amounts of cleaved caspase-3 and p53 protein were detected in infected eyes of KO mice at both time points. (4) Conclusions: caspase-12 contributes to caspase-3-dependent and independent retinal bystander cell death during MCMV retinitis and may also play an important role in innate immunity against virus infection of the retina.

## 1. Introduction

Human cytomegalovirus (HCMV) infects 50 to 80% of humans [1] and primary infection often persists throughout an individual’s lifetime. Although generally asymptomatic in immunocompetent hosts, HCMV infection presents a serious threat to immunosuppressed (IS) or immunonaive individuals [2]. HCMV retinitis is an opportunistic infection and the leading cause of vision impairment and blindness in HIV/AIDS patients without treatment, worldwide [3,4], although less so now than previously. The main causes of visual impairment during HCMV retinitis include macula or optic nerve disease, cataract, retinal detachment, macular edema, immune recovery uveitis, and epiretinal membrane [5,6], during which, necrotic damage to the fovea or optic nerve resulting from CMV infection plays a leading role [4]. Since CMVs are strictly species-specific and HCMV cannot be studied experimentally in vivo, murine cytomegalovirus (MCMV) infection of IS mice has been widely studied, both by ourselves and others, as a model system to decipher the mechanism of HCMV retinitis [7,8,9,10,11,12,13,14,15,16,17,18,19,20]. Increasing evidence suggests that cell death of uninfected bystander retinal cells is an important component of the pathogenesis of cytomegalovirus retinitis and multiple regulated cell death pathways including apoptosis, necroptosis, pyroptosis, and AIF mediated apoptosis-like cell death may be involved in this process [11,14,21,22,23,24,25,26,27,28,29,30,31].

Caspase-12 is a major inflammatory caspase, participating in innate immune responses and inflammation [32,33,34,35,36,37] as well as ER stress-induced cell death [38,39,40]. The role of caspase-12 in innate immune responses against pathogens is unclear and somewhat controversial, possibly due to the variety of pathogens and tissue/cell types that have been used for its study [32,33,34,35,36,37]. For instance, caspase-12 was shown to inhibit mucosal immunity to bacterial infection through suppression of an antimicrobial peptide and the Nod-Rip2-NF-kB signaling pathway [37], as well as inhibition of caspase-1 activation and subsequent IL-1β and IL-18 secretion [32], although the latter remains controversial [33]. Caspase-12 was shown to be activated during respiratory syncytial virus (RSV) and bovine viral diarrhea virus (BVDV) infections [34,35], while activated caspase-12 has been reported to play an important role in controlling West Nile virus (WNV) infection via the pattern-recognition receptor RIG-I and subsequent production of type I interferon [36].

Previous work in our laboratory has demonstrated that caspase-12 is activated during MCMV retinitis [28,41], although its role is presently unclear. One possibility is that caspase-12 could play a significant role in the death of uninfected bystander neuronal cells due to ER stress, since decreased XIAP production in TNF-α deficient mice, resulted in increased caspase-12 activation and apoptosis-like cell death induced by ER stress [28]. In another study, activation of caspase-12 was inhibited substantially in MCMV infected Bax^−/−^ eyes compared to MCMV infected Bax^+/+^ eyes, although more caspase-3–independent death of uninfected bystander retinal cells was observed in Bax^−/−^ compared to Bax^+/+^ eyes [41]. Therefore, in the present study, in order to directly address the role of caspase-12 in MCMV retinitis, we have infected caspase-12 depleted, IS BALB/c mice with MCMV. We report that caspase-12 plays an important role in restricting virus spread in the retina and limiting retinitis during ocular MCMV infection.

## 2. Results

### 2.1. MCMV Spread and Replication

**Figure 1:**
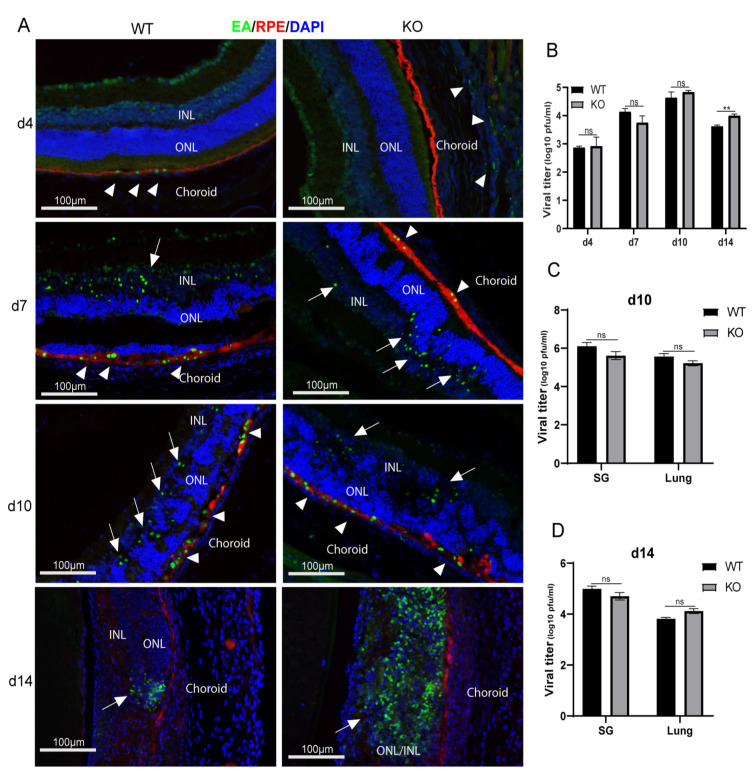
Caspase-12 deficiency promotes virus replication during MCMV retinitis. (**A**) Representative merged photomicrographs of staining for RPE65 (red), MCMV EA (green), and DAPI (blue) in MCMV-infected eyes of IS caspase-12^+/+^ (WT) and caspase-12^−/−^ (KO) mice at day (d) 4, 7, 10, and 14 p.i. (**B**) Titer of MCMV (log10±SEM PFU/eye) in MCMV-injected eyes of WT and KO mice at day 4, 7, 10, and 14 p.i. (**C**) Titer of MCMV (log10 ± SEM PFU/eye) in retinas and non-retina remainders from MCMV-infected eyes of IS WT and KO mice at day 14 p.i. (**D**) Titers of MCMV (log10 ± SEM PFU/organ) in salivary glands and lungs of MCMV infected IS WT and KO mice at day 14 p.i. Data are shown as mean ± SEM (*n* = 8). Statistical analysis by 2-tailed *t*-test. ** *p* < 0.01; ns means of no significance.

**Figure 2:**
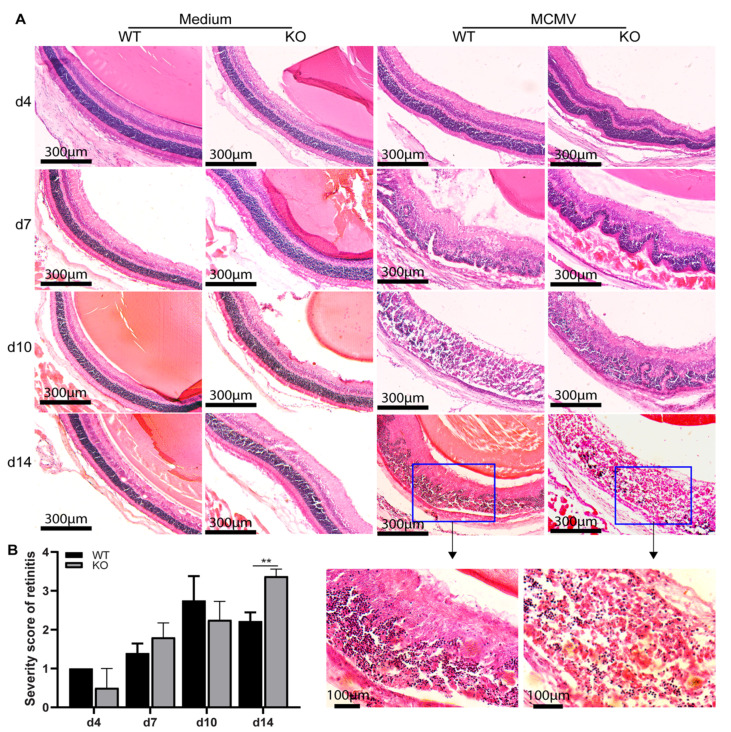
Caspase-12 deficiency exacerbates the severity of MCMV retinitis. (**A**) Representative photomicrographs of H&E staining from MCMV injected eyes of IS WT and KO mice at day 4, 7, 10, and 14 p.i. (**B**) Scoring of retinitis in MCMV-infected eyes of IS WT and KO mice at day 4 (*n* = 3), 7 (*n* = 5), 10 (*n* = 4), and 14 (*n* = 8) p.i. Data are shown as mean ± SEM. Statistical analysis by 2-tailed *t*-test. ** *p* < 0.01.

MCMV early antigen (EA) staining and plaque assay were used to trace virus spread and replication following supraciliary ocular inoculation of MCMV in IS *caspase-12*^−/−^ and *caspase-12*^+/+^ mice. As indicated in Figure 1A, only a few EA-staining, MCMV infected cells were observed in the choroid or RPE of both types of mice at day 4 p.i. (indicated by arrowheads) However, beginning at day 7 p.i., virus spread to the inner retina (ONL and INL indicating the outer and inner nuclear layers of the retina, respectively) was observed (indicated by arrows). Similar numbers of MCMV infected cells and similar virus titers were detected in injected eyes of both types of mice at days 7 and 10 p.i. (Figure 1B). As we have previously observed^24^, MCMV retinal infection was limited at day 14 p.i in wild type BALB/c mice, with fewer EA positive cells and less replicating virus compared to day 10 p.i. In contrast, more replicating virus (Figure 1B) and greater numbers of virus-infected cells (Figure 1A) were detected in the retina of injected eyes of *caspase-12*^−/−^ mice compared to injected eyes of *caspase-12*^+/+^ mice at day 14 p.i. Retinitis was also more severe in *caspase-12*^−/−^ mice at day 14 p.i. H&E staining showed that the average retinopathy score for infected eyes of *caspase-12*^−/−^ mice was significantly higher than that for infected *caspase-12*^+/+^ mice at this time (Figure 2).

To determine if caspase-12 interferes with virus replication in systemic organs and tissues, we analyzed virus titers in the salivary gland (SG) and lung at days 10 and 14 p.i. As shown in Figure 1C,D, there was no significant difference in virus titers of SG and lungs between *caspase-12*^−/−^ and *caspase-12^+/+^* mice.

### 2.2. Interferon Production and Caspase-12 Expression

**Figure 3:**
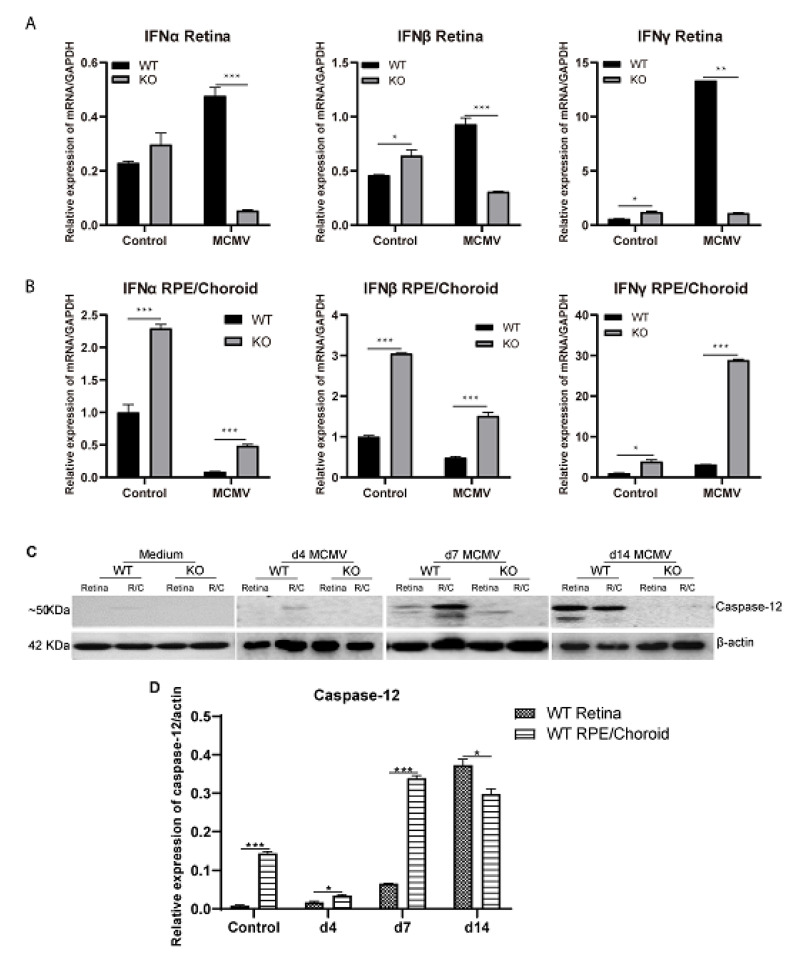
Interferons and caspase-12. (**A**,**B**) Relative expression levels of mRNA for interferons-α, -β and -γ by real-time RT-PCR in the retina (**A**) or posterior eyecups (RPE/choroid, (**B**)) from MCMV-infected eyes of IS WT and KO mice at day 14 p.i. Data are shown as mean ± SEM (*n* = 3). * *p* < 0.05, ** *p* < 0.01, *** *p* < 0.001 by 2-tailed *t*-test. (**C**) Western blot of caspase-12 in retina or posterior eyecups (R/C) from medium-injected and MCMV-injected eyes of IS WT or KO mice at days (d) 4, 7, and 14 p.i. (*n* = 3). (**D**) Ratio of full length caspase-12 (~50 KDa) to β-actin. Data are shown as mean ± SEM. * *p* < 0.05, *** *p* < 0.001 by 2-tailed *t*-test.

Since depletion of caspase 12 was associated with increased virus spread and replication in the retina at day 14 p.i., it is possible that caspase-12 could play a role in defense against retinal virus infection. We hypothesized that caspase-12 might play this role via the production of type-1 interferons based on a previous report that caspase-12 is associated with the production of type I interferons during WNV infection of primary neurons [36]. To test this hypothesis, neural retinas and posterior eye cups consisting of sclera, choroid, and RPE (choroid/RPE) were separated from MCMV injected eyes at day 14 p.i., as well as from medium injected control eyes, and RNA was extracted. mRNA levels of the type I interferons, α and β, as well as the type II interferon γ were analyzed by real-time RT-PCR. Compared to MCMV injected caspase-12^+/+^ eyes, mRNA levels of IFN-α, -β and -γ were significantly lower in the neural retina of MCMV injected caspase-12^−/−^ eyes at day 14 p.i. (Figure 3A), coincident with a more widespread retinal infection in caspase-12^−/−^ mice at this time point. In contrast to the neural retina, mRNA levels of IFN-α, -β and -γ were significantly higher in posterior eye cups of MCMV injected caspase-12^−/−^ eyes at day 14 p.i., compared to MCMV injected caspase-12^+/+^ eyes (Figure 3B).

Previous experiments in our laboratory have demonstrated that caspase-12 is activated in the eye during MCMV retinitis [28,41]. To determine if increased interferon production and limitation of MCMV spread in the retina of wild type mice at later stages of infection is associated with increased in situ caspase-12 expression, eyes were removed from MCMV-infected mice at days 4, 7, and 14 p.i, and neural retinas and posterior eye cups were removed for analysis of caspase-12 expression by Western blot. As shown in Figure 3C, low levels of caspase-12 were detected in RPE/choroid tissues but not neural retinas of uninfected, medium-injected eyes. In contrast, protein levels of caspase-12 were significantly increased at both days 7 and 14 p.i. in the RPE/choroid of MCMV-infected eyes as well as in the neural retina at day 14 p.i., with only a small amount of caspase-12 detected at 7 p.i.

### 2.3. Bystander Retinal Cell Death

**Figure 4:**
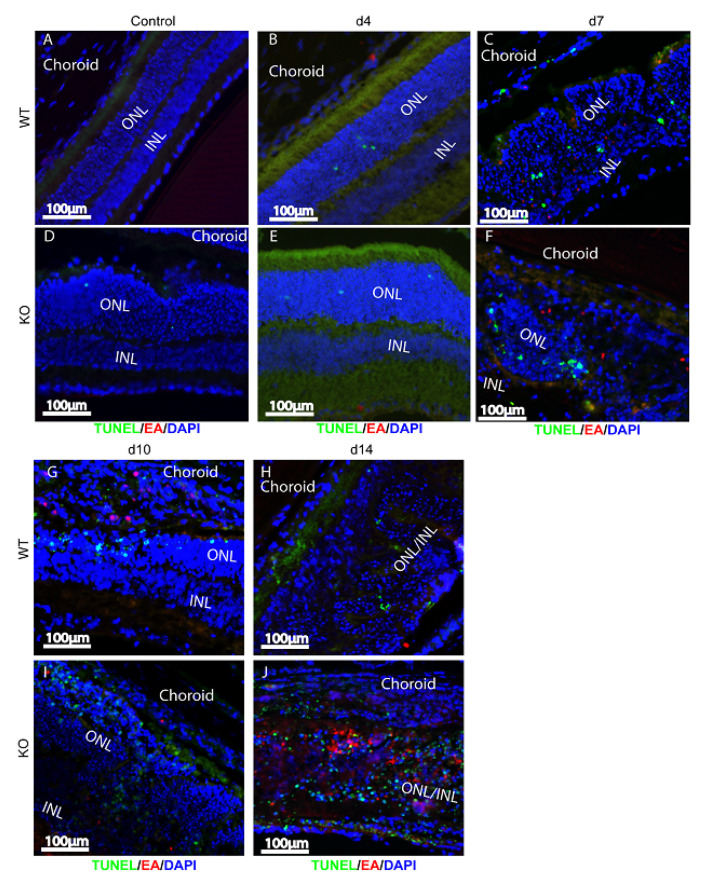
(**A**–**J**) Bystander retinal cell death. Representative merged photomicrographs of TUNEL staining (green), MCMV EA (red), and DAPI (blue) in MCMV-injected eyes or medium-injected controls of WT and caspase-12^−/−^ KO mice at day (d) 4, 7, 10, and 14 p.i. *n* ≥ 3.

Anti-MCMV EA staining and TUNEL assays were used to identify bystander retinal cell death. Similar to previous observations [21,24,28,42], the majority of TUNEL-stained cells were MCMV EA negative, uninfected bystander cells in both MCMV injected caspase-12^−/−^ and caspase-12^+/+^ eyes (Figure 4). Similar numbers of TUNEL-stained cells were observed in the inner retinas of both MCMV injected caspase-12^−/−^ and caspase-12^+/+^ eyes at early times p.i. (Figure 4E,F, compared to Figure 4B,C) whereas increased bystander cell death was observed in caspase-12^−/−^ eyes compared to caspase-12^+/+^ eyes at day 14 p.i. (Figure 4J compared to Figure 4H), possibly due to a wider virus spread and increased virus replication in caspase-12^−/−^ eyes at this time point.

**Figure 5:**
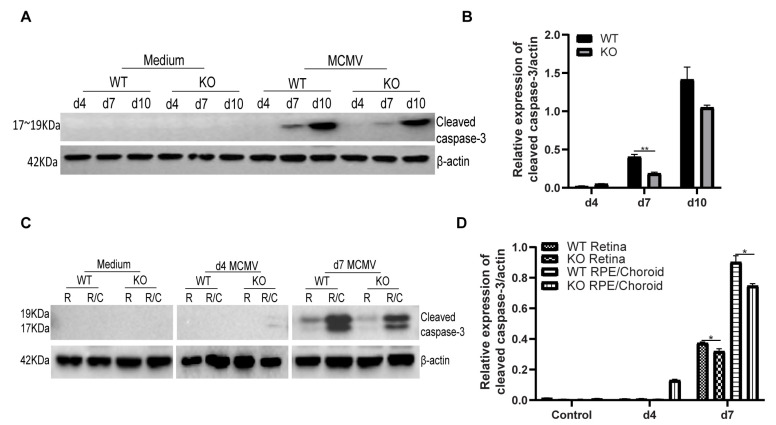
(**A**,**B**) Western blot of cleaved caspase-3 in medium-injected or MCMV-injected eyes of WT or KO mice at day 4, 7, and 10 p.i. (**A**); ratio of cleaved caspase-3 to β-actin (**B**). (**C**,**D**). Western blot of cleaved caspase-3 in the retinas or posterior eyecups (R/C) isolated from medium-injected (control) or MCMV-injected eyes of WT or KO mice at day 4 and 7 p.i. (**C**); ratio of cleaved caspase-3 to β-actin (**D**). Data are shown as mean ± SEM (*n* = 3). * *p* < 0.05, ** *p* < 0.01 by 2-tailed *t*-test.

Since similar virus spread and replication were observed in both caspase-12^−/−^ and caspase-12^+/+^ MCMV injected eyes at days 4, 7, and 10 p.i., eyes were harvested at these time points and prepared for Western blot analysis. Our previous results have shown that both caspase-3-dependent apoptosis and caspase-3-independent apoptosis participate in uninfected bystander retinal cell death [28,41,42]. Therefore, we hypothesized that depletion of caspase-12 might affect the caspase-3-dependent apoptosis pathway, since previous studies have indicated that caspase-12 can directly activate caspase-3 [43]. To test this hypothesis, levels of cleaved caspase-3 were assayed at the above time points by Western blot. As shown in Figure 5A,B, compared to caspase-12^+/+^ mice, less cleaved caspase-3 was detected in injected eyes of caspase-12^−/−^ mice at both day 7 and day 10 p.i.

Interestingly, at day 4 p.i., when only a few TUNEL-stained cells were observed in the inner retina in both caspase-12^−/−^ and caspase-12^+/+^ mice, a small amount of cleaved caspase-3 was observed in injected eyes of caspase-12^−/−^, but not in caspase-12^+/+^ mice. To localize cleaved caspase-3 expression, neural retinas and posterior eye cups were separated from MCMV injected eyes at days 4 and 7 p.i as well as from medium injected control eyes and processed for analysis of cleaved caspase-3 by Western blot. As shown in Figure 5C,D, at day 4 p.i., a small amount of cleaved caspase-3 was detected in the eye cup but not the inner retina of caspase-12^−/−^ mice while no cleaved caspase-3 was observed in either eye cup or retina from caspase-12^+/+^ mice at this early time point. At day 7 p.i., cleaved caspase-3 was detected in eye cups and retinas from both caspase-12^−/−^ and caspase-12^+/+^ mice. Significantly more cleaved caspase-3 was observed in caspase-12^+/+^ retinas than in caspase-12^−/−^ retinas. In addition, more cleaved caspase-3 was observed in caspase-12^+/+^ eye cups than in caspase-12^−/−^ eye cups.

**Figure 6:**
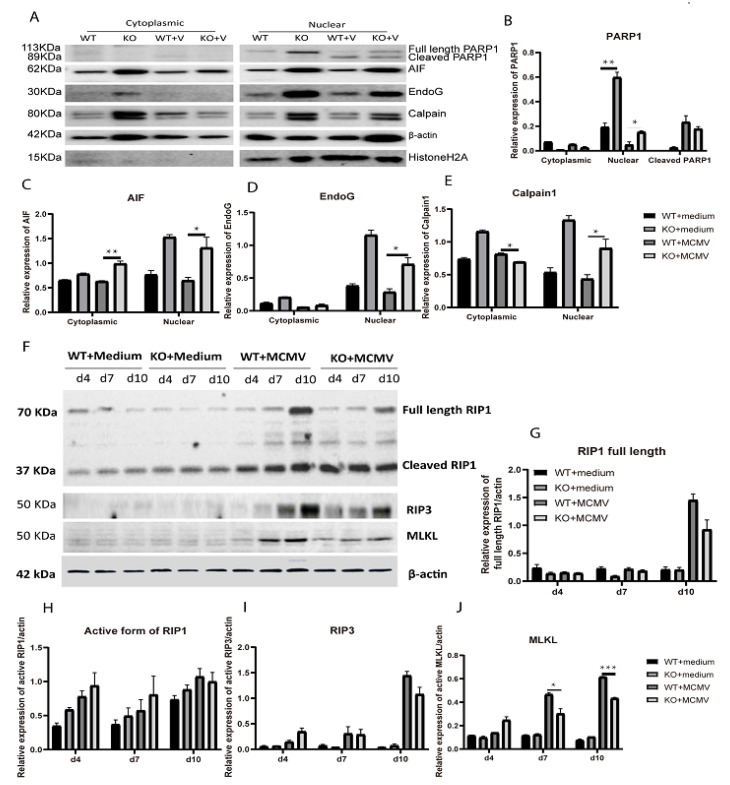
AIF mediated cell death and necroptosis. (**A**–**E**) Western blot showing expression of PARP1, AIF, EndoG and Calpain in the cytoplasmic and nuclear fractions of eyes of mock-infected or MCMV-infected (+v) IS WT or KO mice, at day 7 p.i. (**A**); ratio of PARP1 to β-actin for cytoplasmic fractions and ratio of PARP1 to HistoneH2A for nuclear fractions (**B**); ratio of AIF to β-actin for cytoplasmic fractions and ratio of AIF to HistoneH2A for nuclear fractions (**C**); ratio of EndoG to β-actin for cytoplasmic fractions and ratio of EndoG to HistoneH2A for nuclear fractions (**D**); ratio of Calpain to β-actin for cytoplasmic fractions and ratio of Calpain to HistoneH2A for nuclear fractions (**E**); (**F**–**J**) Western blot of RIP1, RIP3, and MLKL in medium-injected or MCMV-injected eyes of WT or KO mice at day 4, 7, and 10 p.i. (**F**); ratio of full length RIP1 to β-actin (**G**); ratio of cleaved RIP1 to β-actin (**H**); ratio of full RIP3 to β-actin (**I**); ratio of MLKL to β-actin (**J**). Data are shown as mean ± SEM (*n* = 3). * *p* < 0.05, ** *p* < 0.01, *** *p* < 0.001 by 2-tailed *t*-test.

AIF mediated caspase-3-independent apoptotic cell death is a pathway which is activated in many bystander retinal neurons which subsequently die during MCMV retinitis [22,28,30,41]. This involves release of AIF from the mitochondrial inter-membrane space following calpain activation [44,45,46,47] or overactivation of PARP1 [48,49,50,51], before it is translocated to the nucleus where it induces apoptosis-like cell death [44,45]. Caspase 12 could contribute to these pathways since previous studies from other laboratories have indicated that caspase 12 engages in cross-talk with AIF [52] and calpain [53]. To determine if AIF mediated cell death is affected by caspase 12 deficiency, nuclear and cytoplasmic proteins were extracted from caspase-12^−/−^ and caspase-12^+/+^ MCMV injected eyes and medium injected control eyes at days 7 p.i., and the relative protein levels of AIF, PARP1, EndoG, Calpain 1 were analyzed by western blot. As shown in Figure 6A–F, nuclear protein levels of AIF, cleaved PARP1, EndoG, and Calpain 1 were significantly higher in the caspase-12^−/−^ MCMV injected eyes compared to caspase-12^+/+^ MCMV injected eyes. Interestingly, the nuclear protein levels of AIF, cleaved PARP1, EndoG, and Calpain 1 were also higher in the caspase-12^−/−^ uninfected control eyes compared to caspase-12^+/+^ controls eyes although no cell death was observed in control eyes from either caspase-12^−/−^ or caspase-12^+/+^ mice.

The relative levels of RIP3, cleaved RIP1 and MLKL proteins were also analyzed by western blot since necroptosis also contributes to retinal cell death during MCMV retinitis [42] and in several animal models of retinal degeneration [54,55,56,57] while a previous report showed that caspase-12 binds to RIP kinase via its kinase domain [37]. The analyses showed that the amount of MLKL protein was significantly lower in caspase-12^−/−^ MCMV injected eyes compared to caspase-12^+/+^ MCMV injected eyes at both day 7 and 10 p.i., (Figure 6F,J) while no significant differences were noted in levels of either RIP3 or RIP1 between these two group of mice (Figure 6F–I).

### 2.4. p53 Expression

**Figure 7:**
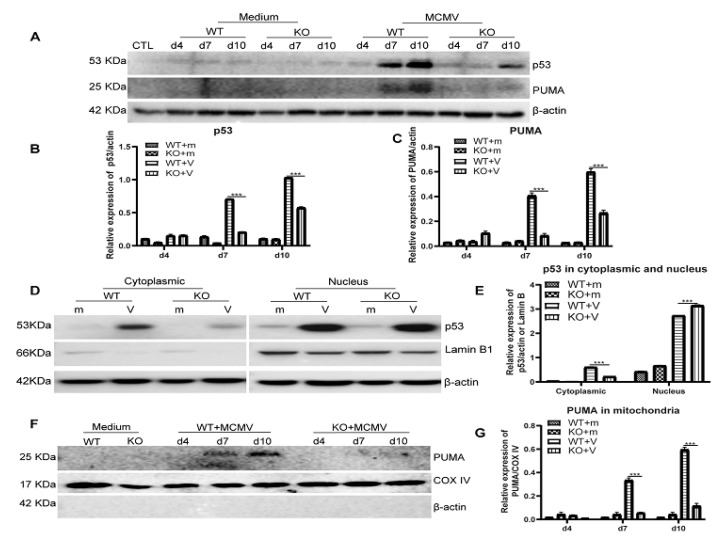
P53 expression. (**A**–**C**) Western blot of p53 and PUMA in medium-injected or MCMV-injected eyes of WT or KO mice at day 4, 7, and 10 p.i. (**A**); ratio of p53 to β-actin (**B**); ratio of PUMA to β-actin (**C**). (**D**–**G**). Western blot showing p53 expression in the cytoplasmic and nuclear fractions of eyes of mock-infected (m) or MCMV-infected (v) IS WT or KO mice at day 7 p.i. Lamin B was the loading control for nuclei (**D**); ratio of p53 to β-actin for cytoplasmic fractions and ratio of p53 to Lamin B for nuclear fractions (**F**); Western blot showing PUMA expression in the mitochondrial fraction of eyes of mock-infected (medium) or MCMV-infected (v) IS WT or KO mice at days 4, 7, and 10 p.i. (**E**); ratio of PUMA to COX IV, the loading control for mitochondria (**G**). (Data are shown as mean ± SEM (*n* = 3). *** *p* < 0.001 by 2-tailed *t*-test.

As a well-known tumor suppressor protein, p53 is activated by various types of cellular stress and regulates several pathways which determine the cell’s fate, whether it be cell cycle arrest or cell death by apoptosis. However, p53 might also be activated by virus infection and play an important role in the innate immune response against virus infection [58,59,60]. To determine if depletion of caspase-12 affects ocular p53 levels following ocular MCMV infection, protein levels of p53 were analyzed by Western blotting. As shown in Figure 7A,B, p53 levels were significantly higher in MCMV injected caspase-12^+/+^ eyes than in caspase-12^−/−^ eyes at both days 7 and 10 p.i. In addition, more p53 protein was present in the cytoplasm of MCMV injected caspase-12^+/+^ eyes than in MCMV-injected caspase-12^−/−^ eyes (Figure 7D,F). In addition, levels of PUMA, an important target protein for p53, were significantly higher in both whole eyes (Figure 7A,C) and mitochondrial fractions (Figure 7E,G) of caspase-12^+/+^ mice compared to caspase-12^−/−^ mice at days 7 and 10 p.i.

## 3. Discussion

Caspase-12, as one of the inflammatory caspases, was first cloned from a murine L929r2 fibrosarcoma cDNA library in 1997 [39]. It is expressed in most organs of BALB/c mice but is found in only a subset of cells in these organs [61], residing principally at the ER where it is cleaved and activated by ER stress [38]. Evidence for caspase-12 involvement in neural cell death has been previously obtained by others. A study by Nakagawa et al. indicated that caspase-12 was involved in ER stress-induced apoptotic signals and contributed to Aβ neurotoxicity in cortical neurons, although it was not essential for apoptosis induced by various types of non-ER stress, such as anti-Fas, staurosporine, anti-Fas plus cycloheximide or TNF plus cycloheximide in liver, kidney, and embryonic fibroblast cells [38]. Other studies have demonstrated that caspase-12 participates in neuronal cell death [62,63] and might contribute to many neuronal diseases including neurodegenerative diseases [64,65], brain ischemic injury [66,67], and retinal diseases [52,68,69]. In addition to ER stress inducing neuronal apoptosis via overexpression of caspase-12 and subsequent activation of caspase-3 [43], cell death could also be triggered by excessive calcium influx, leading to poly (ADP-ribose) polymerase-1 (PARP1) overactivation, and release of AIF [49,70].

Caspase-12 is principally expressed, albeit at low levels, in the RPE/choroid in normal ocular tissue, but during MCMV infection its expression is strongly enhanced while also appearing in the retina at later stages of infection. Although numbers of TUNEL-staining cells in the retina was similar to those in *caspase-12*^+/+^ mice, depletion of caspase-12 resulted in lower levels of cleaved caspase-3 and MLKL in MCMV-infected eyes, consistent with a role for caspase-12 in bystander cell death by caspase-3-dependent cell death and probably by necroptosis.

Previous studies in our laboratory [28], as well as those of others [52], have suggested that caspase-12 also contributes to AIF mediated cell death in the retina. Caspase-12 translocates to the nucleus of dying photoreceptors and has a reinforcing effect on AIF mediated cell death via cross-talk with AIF [52]. In our previous studies, decreased XIAP production and subsequent increased caspase-12 activation and caspase-3-independent apoptosis-like retinal cell death were observed in MCMV-infected, TNF-α deficient mice [28]. Interestingly, depletion of caspase 12 significantly increased nuclear translocation of AIF in MCMV injected eyes as well as in uninfected controls. AIF-mediated cell death was also most likely increased in MCMV-infected *caspase-12*^−/−^ eyes since while numbers of TUNEL-staining cells in the retina were similar in *caspase-12*^−/−^ and *caspase-12*^+/+^ mice, caspase-3-dependent cell death and necroptosis were decreased in MCMV-infected *caspase-12*^−/−^ eyes compared to those in *caspase-12*^+/+^ mice. Further studies are needed to determine if caspase 12 plays a role in limiting AIF translocation from mitochondria to the nucleus.

Our data also suggest a possible role for caspase-12 in the innate immune response against ocular virus infection since depletion of caspase-12 resulted in decreased production of type I interferons. Interestingly, this was restricted to the neural retina and was not observed in non-neuronal ocular tissue, specifically the RPE/choroid. This agrees with previous observations that the exact role of caspase-12 in innate immune responses against pathogens depends on the type of pathogen and tissue/cell type [32,33,34,35,36,37]. The mechanism of type I interferon induction in the retina by caspase-12 remains to be deciphered. One possibility is that it may be related to caspase-12′s role in stimulating pattern-recognition receptor (PRR) pathways, such as RIG-I, as reported for WNV infection of primary neurons [36]. Alternatively, it may be related to the decreased production of p53 in retinas of MCMV-infected *caspase-12*−/− mice, since multiple studies have shown that p53 enhances the production of type 1 interferons via several interferon-regulated pathways [71,72,73,74,75]. Munoz-Fontela C et al. [71] reported that p53 could activate IFN regulatory factor 9, a central component of the IFN-stimulated gene factor 3 complex, while others have shown that p53 activates ubiquitin-like protein interferon-stimulated gene 15 (ISG15) [72], which has been implicated as a central player in the host antiviral response [73]. Toll-like receptor 3 (TLR3) is an important sensor of microbial pathogens and an essential component of innate immune defense against MCMV infection [74]. p53 could also induce expression of TLR3 and subsequent innate immune responses including phosphorylation of interferon regulatory transcription factor 3 [75].

In addition to its role in immune system signaling, p53 is critical for antiviral defense of the host via its ability to mediate apoptosis of virally infected cells, thus limiting the further spread of infectious pathogens [76]. Since levels of ocular p53 were significantly increased following MCMV infection in *caspase-12*+/+ mice, p53 could play a role in defense against retinal MCMV infection by enhancing IFN-dependent antiviral activity. Alternatively, it could mediate apoptosis of virally infected cells although the majority of MCMV-EA positive cells were not stained by TUNEL assay during MCMV retinitis. In addition, p53 might also contribute to bystander retinal cell death. Many viruses, including cytomegalovirus, employ strategies to manipulate antiviral defense mediated by p53 in order to benefit virus spread and replication [58,59,60]. For instance, the HCMV IE2 protein reduces acetylation of p53 and histone H3 which functionally inactivates p53 [77]. However, cytomegalovirus is one of several viruses, which require p53 for their replication [60]. The HCMV genome has 21 potential p53 responsive sites [78]. Compared to wild type cells, HCMV infected p53-negative cells produce fewer infectious virus particles, with delays in virus protein production and trafficking [79]. Therefore, further studies are needed to determine how caspase-12 enhances the production of p53 and the exact role played by p53 in host defense against MCMV retinal infections.

## 4. Materials and Methods

### 4.1. Virus

MCMV strain K181 was originally supplied by Dr. Edward Morcarski (Emory University, Atlanta, GA, USA). Virus stocks were prepared from salivary glands of IS BALB/c mice infected with MCMV as described previously [80]. Viral titer was calculated by plaque assay, providing a quantitative determination of infectious virus concentration. A single infectious virus particle replicates upon infection of a host cell, resulting in production of progeny virus and cell lysis leading to infection of surrounding cells and a repetition of the progeny virus-cell lysis cycle. The result is a visible disruption, or “plaque” in a monolayer of cultured cells.

### 4.2. Reagents and Antibodies

Monoclonal anti-Caspase-12 antibody was purchased from Sigma-Aldrich (St. Louis, MO, USA). Anti-cleaved caspase-3, anti-PUMA, anti-HistonH2A, anti-AIF, anti-RIP1, anti-RIP3, anti-MLKL, anti-p53, anti-COX IV and anti-Lamin B were from Cell Signaling Technology, Inc. (Danvers, MA, USA). Rabbit anti-RPE65 (specific for RPE cells) was from Abcam (Cambridge, MA, USA). The preparation and use of FITC-labeled anti-MCMV EA and biotin-anti-EA has been described previously [24]. Anti-β-actin was from Sigma-Aldrich Corp. (St. Louis, MO, USA). Texas red avidin D, DyLight 594 goat anti-rabbit IgG antibody and DyLight 488 horse anti-mouse IgG antibody were from Vector Laboratories, Inc. (Burlingame, CA, USA). Goat anti-rabbit IgG-HRP, and goat anti-mouse IgG-HRP were from Santa Cruz Biotechnology, Inc. (Santa Cruz, CA, USA). BCA assay kit, NE-PER^TM^ Nuclear and Cytoplasmic Extraction Reagents (78833) and Mitochondria Isolation Kit for Tissue (89801) were purchased from Thermo Fisher Scientific (Waltham, MA, USA). TUNEL assay kit was from Roche (Indianapolis, IN, USA).

### 4.3. Mice

C57BL/6 *caspase-12 ^−/−^* mice were purchased from Mutant Mouse Regional Resource centers (MMRRC) (Stock No: B6.129P2-Casp12^tm1Dgen^/Mmnc) and the derivation of these mice has been previously reported [32]. 6- to 8-week-old *caspase-12^−/−^* and control *caspase-12^+/+^* mice on a BALB/c background were used in all experiments. These mice were obtained by crossing C57BL/6 *caspase-12 ^−/−^* mice with BALB/c mice from Jackson Laboratory (Bar Harbor, ME, USA) for at least 6 generations. All experiments and breeding followed the guidelines of the National Institutes of Health for the care and maintenance of mice and adhered to the ARVO Statement for the Use of Animals in Ophthalmic and Vision Research. The protocols were also approved by the Institutional Animal Care and Use Committee of Augusta University. Anesthesia protocols have been described previously [28]. All mice tested negative by PCR for the rd8 mutation.

### 4.4. Experimental Design

All mice (6 to 8 weeks old) were IS with methylprednisolone acetate as described previously [28,41]. This treatment typically depletes more than 93% of the CD4+ and CD8+ T cells as well as macrophages from MCMV-infected mice, as assayed by flow cytometry of splenocytes [41,81]. Moreover, 5 × 10^3^ PFU of MCMV K181 or medium in 2 ul was injected into one eye via the supraciliary route also as described previously [28,41]. Eyes, salivary glands, and lungs were removed at 4, 7, 10, and 14 days post infection (p.i.) and virus titers were determined by plaque assay. For some experiments, neural retinas and posterior eye-cups (consisting of RPE, choroid, and sclera) were separated from medium or MCMV injected eyes as described previously by our laboratory [30,82]. Additional eyes, neural retinas and posterior eye-cups were prepared for immunohistochemistry, H&E staining, real-time RT-PCR, and Western blots, as described below.

### 4.5. H&E and Immunofluorescence Staining

Infected eyes were isolated and fixed with 4% paraformaldehyde in PBS for 1 h and immersed in 30% sucrose overnight at 4 °C. Eyes were then snap frozen and sectioned on a cryostat. Hematoxylin and eosin (H&E) staining was performed according to standard procedures. For double antibody staining with FITC-anti-MCMV EA and rabbit derived anti-RPE65 or biotin-anti-MCMV EA, all slides were dried for 20 min at room temperature and then washed for 30 min with PBS, followed by permeabilization with 0.1% Triton X-100 in 0.1% sodium citrate for 2 min on ice. Slides were incubated with 10% normal goat serum for 1 h at room temperature and then with primary antibody overnight at 4 °C. Primary antibodies were diluted with PBS containing 0.5% normal goat serum. Following washing with PBS, sections were incubated with DyLight 594 goat anti-rabbit IgG antibody (1:1000) or Texas red avidin D (1:1000) for 1 h at room temperature prior to final washing with PBS.

For TUNEL assay coupled with biotin-anti-MCMV EA, sections were stained initially with TUNEL (in Situ Cell Death Detection Kit, Fluorescein; Roche Diagnostics, Indianapolis, IN, USA) commencing with fixation in 4% paraformaldehyde in PBS for 20 min, following by washing with PBS for 30 min. Slides were then incubated in permeabilization solution for 2 min on ice, rinsed once, and then with 50 ul of TUNEL reaction mixture for 60 min at 37 °C in the dark. Following washing and blocking, sections were incubated overnight at 4 °C with biotin-anti-MCMV EA followed by Texas red avidin D (1:1000) for 1 h at room temperature.

### 4.6. Western Blot

Western blotting of proteins extracted from MCMV-injected or DMEM mock-injected eyes was performed as previously described [28,30]. Mitochondrial proteins were extracted from additional eyes using the Mitochondria Isolation Kit for Tissue (Thermo Fisher Scientific) according to the manufacturer’s instructions. Nuclear and cytoplasmic proteins were extracted from additional eyes using the NE-PER™ Nuclear and Cytoplasmic Extraction kit, also according to the manufacturer’s instructions. Equal amounts of protein were separated by SDS-PAGE followed by transfer onto polyvinylidene difluoride (PVDF) membranes (GE Healthcare, Piscataway, NJ, USA). Blots were blocked with 5% nonfat dry milk for 1 h at room temperature then incubated overnight at 4 °C with primary antibody. Following washing, membranes were incubated with HRP-conjugated secondary antibody for 1 h at room temperature. Immune complexes were detected using chemiluminescence (ECL; GE Healthcare) and exposure with the ChemiDoc XRS+ blot imaging system (Bio-Rad, Hercules, CA, USA). To verify equal loading among lanes, membranes were also probed with anti-β-actin. Band signal intensity was quantified using ImageJ (National Institutes of Health, Bethesda, MD, USA).

### 4.7. Real-Time PCR

RNA was extracted from eyes using TRIzol (Invitrogen, Carlsbad, CA, USA) according to the manufacturer’s instructions. Moreover, 1 µg total RNA was used for cDNA synthesis with the iScript reverse transcription SuperMix (Bio-Rad, Hercules, CA, USA) in a 40 µL reaction mixture, and 1 ul of cDNA was used in a 10 µL PCR amplification reaction. Cycling conditions included 10 min at 95 °C followed by 40 cycles of 95 °C for 15 s, 60 °C for 15 s and 72 °C for 20 s. The specific target gene primers used for cycling are shown in Table 1.

### 4.8. Retinitis Scoring

Scoring of retinopathy severity was according to the system previously described [42,80]. Nine H&E staining sections were evaluated per eye. Changes in the posterior segment, especially the retina, of each section were evaluated microscopically as follows: 0, normal or injection artifact; 1/2, mild atypical retinopathy defined as absence of cytomegaly plus retinal folds involving less than three-quarters of the retinal section; 1, moderate atypical retinopathy defined as absence of cytomegaly plus retinal folds involving more than three-quarters of the retinal section plus photoreceptor atrophy or retinal infiltration by leukocytes involving more than one-quarter of the retina; 2, retinal infection defined as cytomegaly of retinal cells plus partial-thickness retinal necrosis or full-thickness necrosis extending from the ciliary body, but not beyond one-quarter of the retinal section from the ciliary body; 3, necrotizing retinitis defined as cytomegaly plus full-thickness retinal necrosis existing further than one-quarter of a retinal section from the ciliary body or full-thickness retinal necrosis extending from the ciliary body through one quarter of the section; 4, severe necrotizing retinitis defined as cytomegaly with full-thickness necrosis involving the entire retinal section. The average score for all retinal sections of each eye was used to determine the mean retinal score. Data were analyzed using GraphPad Prism 8 (GraphPad Software, La Jolla, CA, USA) to determine if significant differences existed between treatment groups.

### 4.9. Statistical Analysis

All data were expressed as means ±SEM (standard error of mean), where *n* is the number of mice or number of fields used in each experimental group. Statistical significance was determined using either a 2-tailed *t*-test using the GraphPad Prism 8 analysis tool. *p* values < 0.05 were deemed significant.

Interventionary studies involving animals or humans, and other studies that require ethical approval, must list the authority that provided approval and the corresponding ethical approval code.

## Figures and Tables

**Table 1:** Primers used for PCR.

Genes	Primers	
Ifnα	Forward	AACTCCACCAGCAGACAGTG
	Reverse	CATCCAGGCGTAGCTGTTGT
Ifnβ	Forward	AGCGCCAAGCATTCAATGAG
	Reverse	AATCTCTTCCCCACCCCGAA
Ifnγ	Forward	TGCCCAGCAGATCAAGAAGG
	Reverse	TCAGGGGAAATTCCTGCACC
Caspase12 genotyping	GS(E)	GCCAGGAGGACACATGAAAGAGATC
	GS(E,T)	CAGCTGTTCCTGGGAATTGGCAATG
	Neo	GGGTGGATTAGATAAATGCCTGCTCT

## Data Availability

The data presented in this study are included in this published article. All the data can be shared upon request by email.
